# Exosomal MicroRNAs as Potential Biomarkers of Hepatic Injury and Kidney Disease in Glycogen Storage Disease Type Ia Patients

**DOI:** 10.3390/ijms23010328

**Published:** 2021-12-28

**Authors:** Roberta Resaz, Davide Cangelosi, Daniela Segalerba, Martina Morini, Paolo Uva, Maria Carla Bosco, Giuseppe Banderali, Ana Estrella, Corbinian Wanner, David A. Weinstein, Annalisa Sechi, Sabrina Paci, Daniela Melis, Maja Di Rocco, Young Mok Lee, Alessandra Eva

**Affiliations:** 1 Laboratory of Molecular Biology, IRCCS Istituto Giannina Gaslini, Via Gerolamo Gaslini 5, 16147 Genova, Italy; robertaresaz@gaslini.org (R.R.); danielasegalerba@gaslini.org (D.S.); martinamorini@gaslini.org (M.M.); mariacarlabosco@gaslini.org (M.C.B.); 2 Clinical Bioinformatics Unit, IRCCS Istituto Giannina Gaslini, Via Gerolamo Gaslini 5, 16147 Genova, Italy; davidecangelosi@gaslini.org (D.C.); paolouva@gaslini.org (P.U.); 3 Clinical Department of Pediatrics, ASST Santi Paolo e Carlo, Presidio San Paolo, Università degli Studi di Milano, Via Antonio di Rudinì 8, 20142 Milano, Italy; giuseppe.banderali@unimi.it (G.B.); sabrina.paci@asst-santipaolocarlo.it (S.P.); 4 Department of Pediatrics, University of Connecticut School of Medicine, 400 Farmington Ave, Farmington, CT 06030, USA; aestrella@uchc.edu (A.E.); cwanner@uchc.edu (C.W.); weinstein@uchc.edu (D.A.W.); 5 Regional Coordinating Center for Rare Diseases, Presidio Ospedaliero Universitario di Udine, P.zzale SM Della Misericordia 15, 33100 Udine, Italy; annalisa.sechi@asufc.sanita.fvg.it; 6 Department of Medicine, Surgery and Dentistry, Scuola Medica Salernitana, Section of Pediatrics, Università Degli Studi di Salerno, Via Salvador Allende 43, Baronissi, 84100 Salerno, Italy; dmelis@unisa.it; 7 Department of Pediatrics, IRCCS Istituto Giannina Gaslini, Via Gerolamo Gaslini 5, 16147 Genova, Italy; majadirocco@gaslini.org

**Keywords:** microRNA, exosomes, GSDIa, liver, kidney, hepatocellular adenoma, biomarkers

## Abstract

Glycogen storage disease type Ia (GSDIa) is an inherited metabolic disorder caused by mutations in the enzyme glucose-6-phosphatase-α (G6Pase-α). Affected individuals develop renal and liver complications, including the development of hepatocellular adenoma/carcinoma and kidney failure. The purpose of this study was to identify potential biomarkers of the evolution of the disease in GSDIa patients. To this end, we analyzed the expression of exosomal microRNAs (Exo-miRs) in the plasma exosomes of 45 patients aged 6 to 63 years. Plasma from age-matched normal individuals were used as controls. We found that the altered expression of several Exo-miRs correlates with the pathologic state of the patients and might help to monitor the progression of the disease and the development of late GSDIa-associated complications.

## 1. Introduction

Glycogen storage disease type Ia (GSDIa) is a rare disease caused by mutations in glucose-6-phosphatase-α (G6Pase-α), an enzyme expressed in the liver, kidney and intestine, which is fundamental in the terminal steps of gluconeogenesis and glycogenolysis. The lack of a functional G6Pase-α results in the ineffective regulation of glucose homeostasis [1].

GSDIa is a metabolic disorder that causes hypoglycemia, hyperlipidemia, hyperuricemia, and lactic acidemia. The accumulation of glycogen in the liver and kidney causes hepatomegaly and kidney enlargement. Additionally, with time, patients develop serious complications, such as osteoporosis, renal failure and hepatocellular adenomas (HCA), which may progress to hepatocellular carcinomas (HCC). The disease can be managed by a very strict diet to prevent hypoglycemia, consisting of continuous nocturnal gastric drip feeding and/or the frequent oral administration of meals containing complex carbohydrates and uncooked cornstarch [2].

Under dietary therapy, metabolic control can be established in GSD-Ia patients and hypoglycemic episodes can be prevented. Moreover, improved metabolic control reduces the frequency of complications, including HCA and HCC development, ameliorating the prognosis for patients with GSDIa [3,4]. On the other hand, patients with poor metabolic control are still under risk of HCA development [5,6,7]. In the most severe cases liver transplant is the only option. Thus, it is crucial to develop new effective therapies for this disease.

In this respect, the identification of new biomarkers prognostic of the evolution of the disease and diagnostic of tumor formation in livers may serve to develop new pharmacological approaches, especially since GSDIa patients manifest marked variability in the severity of symptoms and long-term complications.

Exosomal microRNAs (Exo-miRs) may represent an important source of disease markers and are being extensively studied because of their mirroring of cellular components. The utilization of blood exosomes as surrogate tissues to diagnose, monitor and predict disease evolution and response to therapy would be a minimally invasive, highly desirable alternative to tissue biopsies [8,9,10].

The use of genetically engineered mouse models can be an efficient way of discovering prognostic markers and can minimize the problems associated with the use of human subjects, such as the low availability of biological samples from patients, especially when rare diseases or children are involved. To this aim, we have previously analyzed the expression of Exo-miRs in the plasma of a liver-specific murine model of GSDIa, LS-*G6pc*^−/−^ mice that we have generated [11]. This animal model could provide an efficient means of discovering the diagnostic markers of hepatic tumors since the liver is the only organ affected in these mice. The results of that study revealed that the altered expression of several Exo-miRs correlated with various pathologic liver states associated with the progression of the disease, among which Exo-miRs discriminated LS-*G6pc*^−/−^ mice with adenomas from LS-*G6pc*^−/−^ mice without adenomas [12].

In the present work, we have analyzed the expression of Exo-miRs in the plasma of GSDIa patients to derive specific biomarkers and prognostic indicators of liver and kidney tissue degeneration and HCA development. We have identified several Exo-miRs that are modulated in patients in comparison with healthy controls and whose altered expression may be correlated with liver disease and tumors. Moreover, we have identified Exo-miRs involved in renal pathology that are deregulated in GSDIa patients, and compatible with the progressive severe kidney disease occurring in GSDIa. In addition, the Exo-miR expression profiles obtained from the patients was correlated with the liver-specific Exo-miR profiles obtained from the LS-*G6pc*^−/−^ mice to derive a common signature that could be specific and prognostic of liver degeneration and connected with the biological pathways associated with tumor development and progression.

We found that several Exo-miRs that are deregulated in GSDIa patients might serve as biomarkers and thus help to monitor the progression of the disease and the development of late GSDIa-associated complications.

## 2. Results and Discussion

### 2.1. Exo-miRs Expression Profiling of GSDIa and CTRL Plasma Exosomes

**Figure 1:**
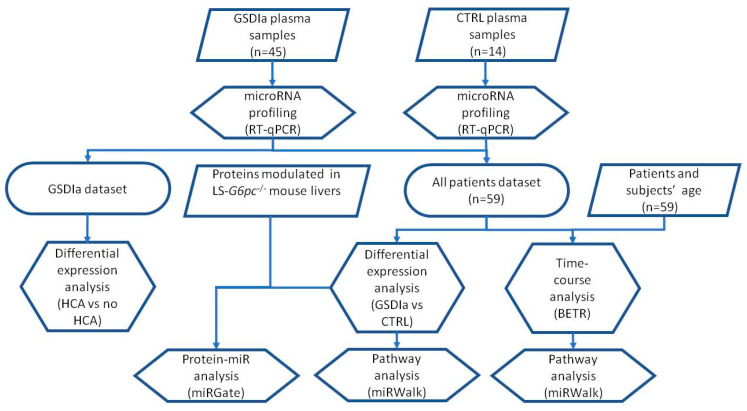
Schematic representation of the whole bioinformatic strategy used in the study. The Exo-miR representation profiles of plasma exosomes of 45 GSDIa patients and 14 CTRL subjects were measured via ViiA 7 RT-qPCR. Differential expression analysis assessed any significant modulation of the Exo-miRs between GSDIa patients and CTRL subjects or GSDIa patients characterized by the presence/absence of hepatic adenomas. The representation profile of GSDIa patients and CTRL subjects over three distinct age groups was compared using BETR method. Pathway analysis carried out on significantly modulated Exo-miRs identified the most significantly altered biological processes and pathways using the MirWalk tool. The potential regulatory activity of Exo-miRs in GSDIa patient liver was then evaluated using a set of proteins previously identified by our group to be modulated in LS-*G6pc*^−/−^ mouse livers using the miRGate tool. Trapezoidal boxes around a text in the workflow indicate input data, hexagonal boxes indicate analyses, and smooth rectangular boxes indicate datasets.

We compared the Exo-miR expression profiles of 45 GSDIa patients versus 14 control (CTRL) subjects in order to identify those miRNAs specific for the pathologic manifestations of GSDIa and/or for its evolution. The analysis workflow is summarized in Figure 1. GSDIa patients’ ages ranged from 6 to 63 years. CTRL subjects’ ages matched that of the GSDIa patients for comparability between groups. Twenty GSDIa patients developed HCA, whereas, as expected, none of the CTRL patients developed HCA. The characteristics of the GSDIa patients used in the study are summarized in Table 1.

**Figure 2:**
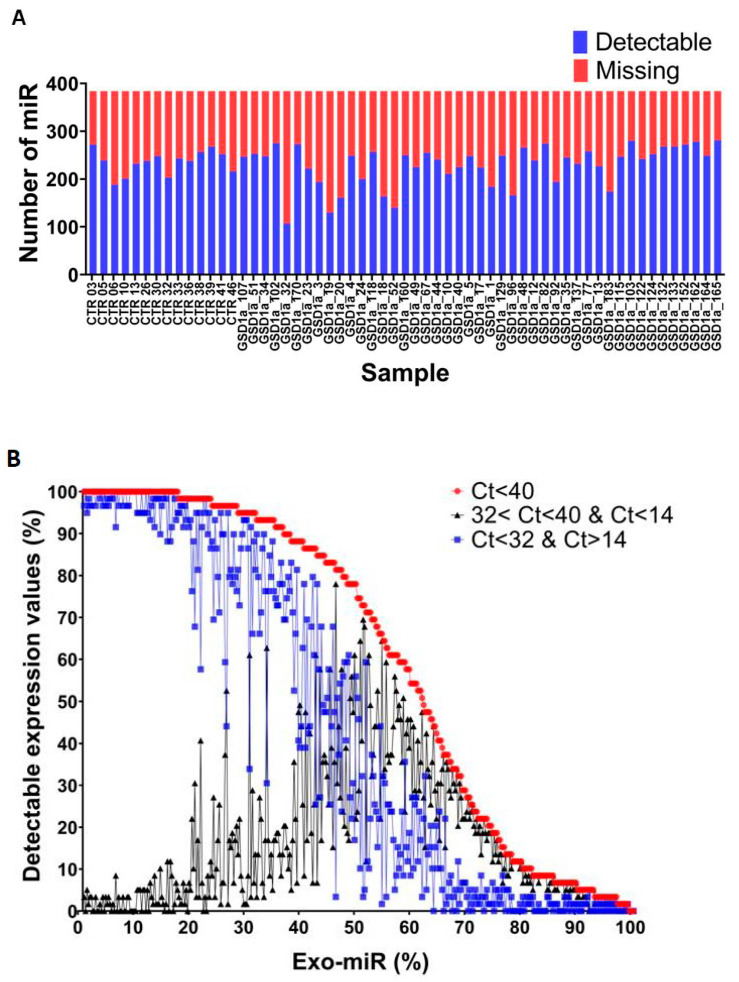
Quality assessment of Exo-miR expression profiles. The plots in panels (**A**) and (**B**) show the distribution of detectable and missing Ct values across samples. Panel (**A**) shows a stacked column chart for visualizing the proportion of detectable (blue column) and missing (red stacked column) Ct values across the samples. Sample identifiers are shown on the x axis. The number of microRNAs is shown on the y axis. Panel (**B**) shows the percentages of Exo-miRs and Ct detectable values for three levels of reliability of the Ct values. Reliable and unreliable Ct values are colored in red. Reliable Ct values are colored in blue. Unreliable Ct values are colored in black. Curves are sorted in decreasing order of the percentage of miRNAs with Ct < 40 values. The legend is displayed in the top-right part of the chart.

The RNA was isolated from plasma exosomes, reverse transcribed, pre-amplified and used to arrange for each sample a human microRNA array card for the measurement of the expression of 384 targets by the qRT-PCR. A visual inspection of the percentage of raw cycle thresholds (Ct) and missing values across all sample profiles indicated a comparable percentage of Ct and missing values across the samples (Figure 2A). Raw data were processed using the PIPE-T tool, a bioinformatics tool specialized in the analysis of RT-qPCR expression data [13] (see Materials and Methods). A Ct between 14 and 32 were considered to be reliable in accordance with the manufacturer’s instructions. PIPE-T identified a total of 9775 reliable Ct values and 3864 unreliable values. Distribution analysis based on the number of reliable and unreliable Ct values for all Exo-miRs showed that ~45% of Exo-miRs had a higher number of samples with reliable Ct values with respect to unreliable ones (Figure 2B). This indicates that the Exo-miR profiles of GSDIa patients and CTRL subjects is a potential reliable measurement of Exo-miR expression.

Visual inspection of the distribution of the most reliable expression values across the samples shows a clear heterogeneity among Exo-miR expression profiles (Supplementary Figure S2A), which may be caused by the presence of unwanted technical variability in the data [13]. The global mean [14] was used to normalize per-sample Exo-miR expression profiles and was effective at reducing unwanted technical variability (Supplementary Figure S2B). Furthermore, technical variability reduction was significant (Kolmogorov–Smirnov *p*-value < 0.05, Supplementary Figure S2C).

Missing values are difficult to handle using standard statistical analysis [13]. We used the Mestdagh method [13] for imputing missing values for samples that had at most 20% of their values missing. Data relabeling, normalization, filtering and imputation allowed the selection of expression values of sufficiently high quality for a total of 103 Exo-miRs that were used in subsequent analyses (data not shown).

### 2.2. Deregulation of Exo-miRs in GSDIa Patients

In order to identify specific deregulated Exo-miRs that could represent new potential biomarkers of disease development and progression, we first analyzed the expression levels of Exo-miRs in GSDIa patients and compared them with the expression levels of Exo-miRs in CTRL subjects. Our analysis identified six upregulated and four downregulated Exo-miRs in GSDIa compared to CTRL patients (Table 2). Violin plots, showing the differential expression of each modulated miR, are reported in Supplementary Figure S3. Of these, several, including miR-483-5p [15], mir122-5p [16,17], miR-454-3p [18,19], miR-376c-3p [20,21], miR-145-5p [22,23], miR-324-5p [24,25] and miR-342 [26], are considered to be biomarkers of HCC and are involved in HCC growth, metastasis or resistance to chemotherapy. Moreover, miR-122-5p, miR-103-3p and miR-27b-3p have been associated with the signaling pathways relevant in glucose and lipid metabolism. In fact, miR-122-5p is associated with liver steatosis and fibrosis and can be used as biomarker for fatty liver disease [27]. The elevated expression of exosomal miR-122 correlates with obesity and increased triglyceride levels and its inhibition reduces cholesterol and hepatic fatty acids synthesis in mice [28]. Treatment with fenofibrate upregulates miR-103-3p, ameliorating insulin sensitivity in obese mice [29]. Finally, a recent study by Ma et al. [30] showed that the family of miR-27 regulates metabolic genes, including those involved in gluconeogenesis, glycolysis, lipid biosynthesis and lipolysis.

We also found that some of these microRNAs are relevant in kidney injury, failure and chronic disease. In particular, these microRNAs have been associated with diabetic kidney disease, one of the major microvascular complications in patients with type 1 or type 2 diabetes, which represents the primary cause of end-stage renal disease. miR-483-5p has diagnostic value in diabetic nephropathy and can protect human proximal renal tubular cells from the apoptosis and inflammation induced by high glucose [31]. The serum levels of miR-122-5p correlate with the albuminuria, glomerular filtration rate, blood glucose and lipid profiles in patients with diabetic kidney disease and type 2 diabetes mellitus [32]. miR-342-3p was reported to be involved in the pathways related to diabetic kidney disease pathogenesis, such as apoptosis, fibrosis and extracellular matrix accumulation, and has also been reported to suppress the progression of diabetic kidney disease by inducing the degradation of SOX6, a member of the SOX family of transcription factors, thus representing both a potential biomarker of this disease and a novel therapeutic target in the treatment of diabetic kidney disease [33,34]. miR-27b-3p inhibits renal fibrosis, a pathologic aspect of chronic kidney disease, and thus epithelial-to-mesenchymal transition, by downregulating STAT1, α-SMA and collagen III [35], and has been identified in urine extracellular vesicles as a biomarker of diabetic nephropathy [36]. Finally, miR-150-5p plays a reno-protective role in mice affected by diabetic nephropathy through targeting *SIRT1* and is detectable in the serum and urine of patients with diabetic nephropathy [37].

The finding of Exo-miRs involved in renal pathology is very relevant considering that progressive severe kidney disease is a major long-term pathological manifestation in GSDIa. In fact, the pathological manifestations of the life-threatening kidney disease in GSDI overlap with diabetic nephropathy. Both GSDI and diabetic nephropathy start with a long period of silent glomerular hyperfiltration, followed by the development of microalbuminuria, proteinuria and, eventually, renal failure [38]. Diabetic nephropathy is a complex, multifactorial disease and risk factors include hypertension, dyslipidemia and polymorphisms in angiotensin converting enzyme (ACE) genes. Treatment with ACE-inhibitors is effective at decreasing glomerular hyperfiltration, but not at improving microalbuminuria and proteinuria [39]. Thus, deregulated Exo-miRs may represent potential disease markers for, and contributors to, both the liver and kidney pathological manifestations caused by GSDIa.

We then compared the Exo-miR expression levels in GSDIa patients with HCA with the Exo-miRs expression levels in GSDIa patients without HCA. Differential expression analysis identified three upregulated and two downregulated Exo-miRs in GSDIa patients with HCA versus GSDIa patients without HCA (Table 2). Violin plots, showing the differential expression of each modulated Exo-miR, are reported in Supplementary Figure S3. The deregulation of some of these miRs was previously considered to be a contribution to the pathogenesis of HCC, including miR-150-5p, miR-221-3p and miR-203-3p. In particular, it was reported that miR-221-3p is upregulated in liver cancer tissues and cells and that this is associated with infiltration and poor prognosis. In fact, miR-221-3p can promote the viability, migration and invasion of HCC cells by suppressing DNA repair enzyme MGMT transcription and translation. In addition, the overexpression of miR-221-3p promoted liver cancer cell proliferation and invasion in vitro [40,41]. The overexpression of miR-221-3p is also associated with the inhibition of apoptosis, the activation of the TGF-β, Wnt/β-catenin and mTOR signaling pathways, cell migration, invasion, and the formation of a more aggressive tumor phenotype [42,43]. miR-203-3p overexpression markedly inhibits the proliferation, invasion and metastasis in HCC, through suppressing the expression of *KI67* and *CAPNS1* and its overexpression reverses the epithelial–mesenchymal transition induced by hepatectomy through the targeting of *IL-1Β, SNAIL1* and *TWIST1* [44]. Another study hypothesized that the downregulation of miR-203-3p may contribute to carcinogenesis by activating Abce1, a protein that is overexpressed in some malignant cells, including melanoma cells, and some drug-resistant cancer cell lines [45]. Moreover, miR-203-3p is involved in other hepatic malignancies, such as hepatoblastoma [46], a primary pediatric malignant liver tumor, where it is downregulated, and its inhibition promotes liver fibrosis [47].

Therefore, the altered expression of several Exo-miRs may be correlated with pathologic liver conditions and might help to discriminate between affected patients during the progression of the disease and the development of HCA and HCC.

### 2.3. Age-Dependent Modulation of Exo-miRs in GSDIa Patients

**Figure 3:**
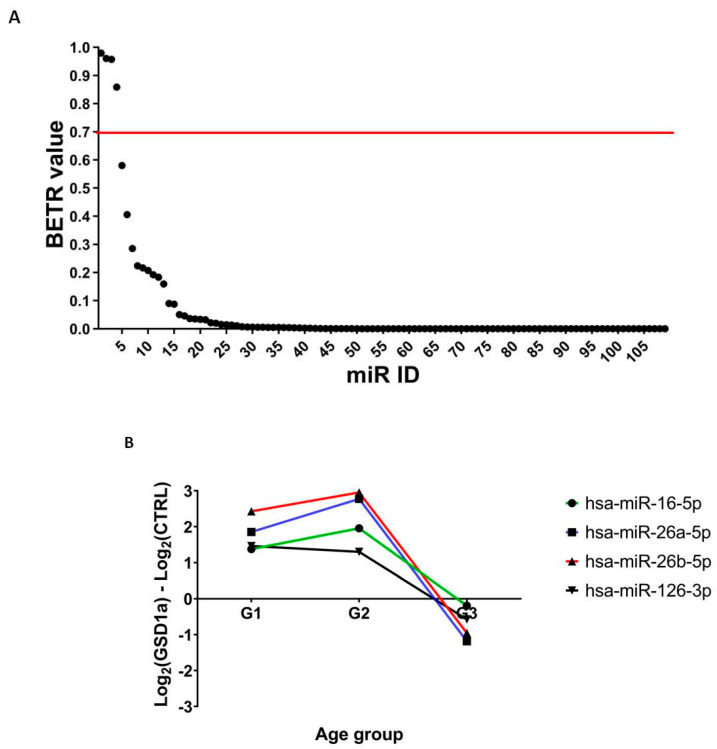
Time-course analysis reveals an age-dependent modulation of Exo-miR expression in GSDIa patients. The plots in (panels **A**,**B**) show the results of the time-course analysis between GSDIa patients and CTRL subjects using the BETR method. Panel **A** reports the BETR values of all Exo-miRs sorted in decreasing order. Exo-miRs with a BETR value greater than 0.7 provided the best evidence for differential expression and were considered to be significantly modulated. The red line displays the threshold value to visually differentiate significantly and not significantly modulated Exo-miRs. Panel **B** shows the log2 fold change value of the four significantly age-dependent and differentially represented Exo-miRs between GSDIa patients and CTRL subjects according to the BETR method. GSDIa patients and CTRL subjects were grouped into three age groups: G1 (6–10); G2 (11–20); G3 (21–63). The group identifier is shown in the x axis. Different colors and symbols were used in the line plot to differentiate the four modulated Exo-miRs. The legend is reported on the right side of the plot.

The age-dependent modulation of Exo-miRs might be instrumental to study the evolution of the disease, and to find biomarkers prognostic of HCA and HCC and the onset of GSDIa. Patients were assembled into three groups according to their age at the time of sample collection (1–10, 11–20 and 21–60 years) to reflect the different stages of disease progression. The levels of expression of the 103 Exo-miRs were examined in the GSDIa patients across the age groups using the BETR method. For each Exo-miR, BETR calculated a numeric value indicating the probability of the differential expression of Exo-miRs in the dataset [48]. Exo-miRs that obtained BETR value greater than 0.7 provide the best evidence for differential expression [48]. The BETR values of all Exo-miRs are plotted in decreasing order in Figure 3A. The level of expression of four Exo-miRs was significantly modulated in GSDIa patients compared with the CTRL subjects in all age groups. The levels of expression of miR-16-5p, miR-26a-5p, miR-26b-5p and miR-126-3p decreased over time, starting from an upregulation in younger patients and becoming downregulated in the older patients. The plots in Figure 3B show the log2 fold change value for the four significantly differentially represented Exo-miRs identified by the BETR method for GSDIa patients and CTRL subjects grouped by age. These four microRNAs have all been reported to be biomarkers of HCC. Gain-of-function studies showed that miR-16-5p downregulation promotes HCC progression and correlates with adverse clinical features and the poor prognosis of HCC patients. The downregulation of miR-16-5p seems to be due to the sponging effect of overexpression of the long non-coding RNA AGAP2-AS1, caused by hypoxia, and linked to the over-expression of *IGF1R*, which is highly expressed in tumor tissues and a target of miR-16-5p [49,50]. Similarly, the downregulation of miR-26a-5p and miR-26b-5p was correlated with a high level of expression of *IGF2* in patients with hepatitis B virus-related HCC [51,52]. miR-126-3p was found to inhibit HCC metastasis and angiogenesis by targeting *LRP6* and *PIK3R2* [53]. The association of miR-126-3p with invasion and metastasis in HCC was later confirmed through a bioinformatic analysis followed by in vitro experimental validation [54]. Interestingly, miR-16-5p and miR-26b-5p are also involved in nephropathy and diabetic kidneys. miR-16-5p was found to be downregulated in a group of patients with severe diabetic kidney disease, while miR-26b-5p has been associated with acute kidney injury [55] and hypertensive nephropathy [56].

These findings indicate an age-dependent modulation of Exo-miR expression as the GSDIa patients became older and suggest that alterations of these microRNAs are compatible with their involvement in the long-term complications associated with GSDIa.

### 2.4. Enrichment of Specific GO Biological Processes and KEGG Pathways in the Exo-miR Expression Profile of GSDIa Patients

We performed a pathway analysis based on the microRNAs’ target genes using gene ontology (GO) processes and the Kyoto Encyclopedia of Genes and Genomes (KEGG) pathway ontologies. Pathway analysis was carried out for each significant Exo-miR using the MirWalk tool [57]. Each significantly enriched pathway was associated with its regulating microRNA (Table S1). For the Exo-miRs significantly modulated in GSDIa patients, MirWalk identified 11,260 targets. Pathway analysis showed the significant enrichment of several GO biological processes and KEGG pathways (*p*-value < 0.05; Table S1). Among them, we observed an enrichment of the target genes associated with pathways of glucose, glycogen, and lipid metabolism, insulin, Wnt_signaling, hypoxia, cytokines and interleukins, autophagy, mitochondria and calcium ion transport (Table S2). This is particularly interesting because all of these pathways are deregulated in GSDIa and contribute to the clinical and pathological characteristics of the disease. For example, it is reported that GSDIa patients display impaired hepatic autophagy and an inflammatory environment, potentially leading to hepatic tumor development. In fact, Gautam et al. [58] show that autophagy impairment in GSDIa is caused by the impaired signaling of *SIRT1*, *FoxO3a*, *AMPK*, and *PPAR-α*, and most of these genes are targets of the deregulated microRNAs we identified. Moreover, a study by Rossi et al. [59] suggests the presence of mitochondrial impairment in GSDIa patients. They hypothesize that the G6Pase deficiency may lead to mitochondrial impairment in the presence of a high carbohydrate diet, linking the mitochondrial dysfunction with insulin resistance. In our previous work, we analyzed the proteomic profile of our mouse model of GSDIa and found that many of the proteins correlated with hypoxia, inflammation and enhanced hepatic glycolysis and gluconeogenesis were over-represented. Furthermore, we found the enrichment of the target genes involved in cellular proliferation and tumor development, including *SMAD*, *FoxO*, *mTOR* and *Notch* signaling pathways (Table S2). Finally, some of the target genes of miR-145-5p, miR-150-5p, miR-483-5p and miR-103-3p act as tumor suppressor or oncogenes in HCC and are involved in carcinogenesis (Table S2). Therefore, these microRNAs may be considered to be new potential therapeutic targets to prevent or counteract the development of liver tumors in GSDIa.

### 2.5. Overlapping between MicroRNA Targets and Proteins Modulated in LS-G6pc^−/−^ Mouse Livers

To identify potential biomarkers of the pathophysiology of the GSDIa-affected liver we have recently analyzed the plasma exosomes of a murine model of GSDIa in an LS-*G6pc*^−/−^ mouse [11] to uncover the modulation of the microRNA expression associated with the disease [12], as well as the proteomic expression profile in the liver of the same mouse model [60].

On the basis of those findings, we have here evaluated whether the Exo-miR modulation of expression found in GSDIa patients would overlap with that obtained in mice and thus allow us to derive a common signature of Exo-miRs that could be specific and prognostic of liver degeneration and HCA development in this disease.

We first compared the Exo-miRs found modulated in humans with those modulated in mice. miR-145-p and miR-203-3p, biomarkers of HCC and involved in HCC growth [1,47,61], were found to be similarly modulated in both humans and mice. miR-145-p, found to be downregulated in LS-*G6pc*^−/−^ mice with tumors, is involved in the signaling pathways associated with HCC, including Wnt, TGFβ, and Ras, interacts with circular RNA in HCC [62] and is one of the integrated signatures of the 13 microRNAs identified in HCC [43]. miR-203-3p has an oncosuppressor activity that impacts on the growth, aggressiveness and prognosis of HCC [63], and was found to be downregulated at different time points during disease progression in the LS-*G6pc*^−/−^ mice in comparison to wild-type mice.

We then evaluated whether the Exo-miRs modulated in patients would regulate genes expressing the proteins previously found differentially represented in the LS-*G6pc*^−/−^ mice livers and associated with specific biological pathways [60].

To this end, we first converted the symbols of the proteins modulated in LS-*G6pc*^−/−^ mice into human genes and then used the miRGate database [61] to extract a list of predicted or experimentally validated human Exo-miRs regulating the expression of these genes. Lastly, we extracted from this list the Exo-miRs we had found modulated in GSDIa patients. This analysis identified several microRNAs able to regulate the genes expressing the proteins differentially represented in the proteomic profile of the LS-*G6pc*^−/−^ mice, including protein sets related to response to hypoxia, glucose and lipid metabolism, and the inflammatory and immune responses (Table 3).

In particular, we identified CD163, the acute phase-regulated scavenger receptor, involved in the clearance of hemoglobin/haptoglobin complexes by macrophages, as a target of miR-19a-3p, the mannose-binding protein C, MBL2, as a target of miR-19a-3p, miR-145-5p, and miR-203a-3p, and the complement components C3 and C5, which play a fundamental role in the activation of the complement system, as targets of miR-27b-3p and miR-19a-3p, respectively. Thus, the Exo-miRs we have identified modulated in GSDIa patients may be involved in the regulation of the process of tissue inflammation and macrophage polarization, and therefore associated with tumor progression, similarly to what was found in the liver of the GSDIa animal model.

We then identified several proteins implicated in glucose and lipid metabolism, whose coding genes are targets of the Exo-miRs modulated in the GSDIa patients and previously found to be over-represented in LS-*G6pc*^−/−^ mouse livers. In particular, ACACA and ACACB are involved in fatty acid biosynthesis, while FDPS, HMGCS1, MVD and TM7SF2 are involved in cholesterol biosynthesis, and GOT, GPT, IDH4 and LDHA are involved in 2-oxocarboxylic acid metabolism.

Finally, several of the proteins involved in hypoxia that were found to be over-represented in mouse livers were found to be targets of GSDIa patients’ Exo-miRs. Of the 24 proteins that mostly contributed to the enrichment of the HALLMARK_HYPOXIA protein set in the LS-*G6pc*^−/−^ mice, 13 were found to be predicted targets of the Exo-miRs modulated in humans. Of these, three are enzymes involved in the lactic acid production by anaerobic glycolysis, including LDHA, PKLR and GAPDH.

Our analysis reveals that the Exo-miRs that are significantly modulated in GSDIa patients regulate genes connected with the biological pathways previously identified by the proteomic analysis of LS-*G6pc*^−/−^ mice livers as being associated with the reprogramming of glucose-6-phosphate and with tumor development and progression.

## 3. Materials and Methods

### 3.1. Patients Blood Sample Collection

GSDIa patients between 6 year and 63 years of age, undergoing periodic evaluation, have been included in the study. As a control, age-matched healthy donors have been enrolled. A detailed clinical and laboratory examination of the control subjects was carried out to rule out infections, inflammation, chronic diseases and liver and kidney altered functional parameters. Written informed consent from the patients or their legal guardians was obtained prior to sample collection. Plasma samples and isolated microRNAs have been provided by the BIT-Gaslini Biobank of the IRCCS G. Gaslini, Genova, Italy and the Biobank of Glycogen Storage Disease Laboratory at UConn Health Center (Farmington, CT, USA).

### 3.2. Exosome Isolation, MicroRNA Purification and Quantitative Real-Time PCR (qRT-PCR)

Plasma was obtained by centrifuging blood samples at 1500× *g* for 10 min at room temperature and was stored at −80 °C for exosomes isolation. Exo-miRs were prepared from 500 μL of plasma, after centrifugation at 16,000× *g* at 4 °C to eliminate cellular debris, utilizing the ExoRNeasy Midi kit (Qiagen Italia, Milano, Italy), according to the manufacturer’s instructions. Quality control and microRNA evaluation were determined with the Agilent 2100 Bioanalyzer, using the small RNA assay (Agilent Technologies Spa, Milan, Italy). To collect intact exosomes, the exoRNeasy Serum/Plasma Midi kit protocol was used: in the last step of the process, QIAzol was substituted with 150 μL of Buffer XE and the particle size was evaluated using the zetasizer nano ZS90 particle sizer (Malvern Instruments, Worchestershire, UK). As determined by dynamic light scattering analysis, isolated vesicles had the typical size range of exosomes, ranging between 30 and 120 nm (Supplementary Figure S1).

Exo-miRs were analyzed using the TaqMan Array Card Technology. Briefly, 50 ng of RNA were reverse transcribed with the TaqMan^®^microRNA Reverse Transcription Kit and the Megaplex^TM^RT primers Human Pool A (Thermo Fisher Scientific, Monza, MB, Italy). cDNA was pre-amplified with TaqMan^®^PreAmp Master Mix and MegaplexTM Pre-Amp primers Human Pool A. The pre-amplified product was diluted according to the manufacturer’s instruction and mixed with 450 μL of TaqMan^®^Universal Master Mix II, No UNG (ThermoFisher Scientific, Monza, MB, Italy), and 441 μL of nuclease-free water. The microRNA profiling was performed with the ViiA^TM^ 7 Real-Time PCR System on the TaqMan^®^Array human microRNA A card (Thermo Fisher Scientific, Monza, MB, Italy), enabling the quantification of 381 human microRNAs.

### 3.3. Bioinformatic Procedures and Statistical Analysis

RT-qPCR data processing, categorization, normalization, filtering, imputation and differential expression were performed using the PIPE-T Galaxy tool [13]. The Ct values falling within the range of 14–32 were categorized as reliable values as recommended by the guidelines of the manufacturer. Global mean normalization was used to reduce any technical variability introduced in the data by the RT-qPCR experiments. Noise reduction was assessed by the Kolmogorov–Smirnov test. Only Exo-miRs with ≤20% of missing values were retained for the analysis to reduce the bias introduced by imputation. The Mestdagh method was used to assign a numeric expression value to missing values. The rank product method was used to identify significant differentially expressed microRNAs. Pathway analysis was performed for both predicted and validated targets of an Exo-miR using mirWalk version 3.0 [57] and carried out using GO and KEGG gene set collections. For time-course analysis, patients were grouped into three groups according to their age at the time of sample collection (6–10, 11–20 and 21–63 years) to reflect different stages of disease progression, and the analysis was carried out using the BETR R package [13]. To control the expected number of false-positive findings, we set up a maximum false discovery rate (FDR) of 5%. In order to focus on the most reliable age-dependent modulated Exo-miRs, we considered an Exo-miR to be significant if the differential expression probability was greater than 0.7. The significance of the difference of the number of missing values between GSDIa patients and CTRL subjects was calculated using Student’s unpaired *t*-test. Statistical analysis and plots were carried out using GraphPad Prism version 8.0 for Windows). miRGate, a curated database of human, mouse and rat miRNA–mRNA targets [15], was used with the proteins modulated in the LS*-G6pc^−/−^* mouse livers [16] to identify microRNAs potentially targeting genes coding for those proteins. Human gene symbols associated with proteins were detected using the HGNC database [17] (https://www.genenames.org/ (accessed on 21 October 2021)). Candidate microRNAs were compared with the significantly modulated Exo-miRs in the GSDIa patients identified in this study to assess the association between liver degeneration and Exo-miR deregulation in GSDIa patients.

## 4. Conclusions

In our study, we investigated microRNA expression profiles in the circulating exosomes of GSDIa patients in order to identify the Exo-miRs that are relevant as biomarkers of the pathological manifestation and progression of disease. The results of our studies delivered several microRNAs relevant as biomarkers to various statuses of the disease. We could identify the microRNAs associated with liver disease and the metabolic alterations of glucose and lipid pathways. We also found the deregulation of microRNAs relevant in liver tumor development and, finally, several microRNAs whose altered expression has been associated with diabetic and chronic kidney diseases.

These results are important because they highlight the potential of plasma exosomes to be surrogate tissues to study the development of the disease and the onset of serious complications. The existence of a communication network between pathologic tissues and their environment through the shedding of exosomes has been shown in several diseases, including cancer, rare diseases and tissue degeneration. For example, exosomes can be readily detected in higher concentrations in the serum and plasma of cancer patients. Thus, the utilization of blood exosomes as surrogate tissue to diagnose, monitor and predict disease evolution and response to therapy constitutes a less invasive and highly desirable alternative to biopsies.

The availability of an animal model is indispensable to compensate for the limitations of testing patients with rare diseases. The LS-*G6pc^−/−^* mouse has proven to be essential in the present and previous studies, since it reproduces all the pathological characteristics of the GSDIa liver, including hepatomegaly, glycogen accumulation, hepatic steatosis, progressive hepatic degeneration and liver tumor development.

The profiles of blood Exo-miRs levels in control and LS-*G6pc^−/−^* mice has been determined utilizing the same molecular and informatics tools described in the present manuscript and have now been used to identify the specific manifestations of liver Exo-miR contributions, since in our experimental model only the liver is affected. On the other hand, we also identified a cluster of Exo-miRs, involved in kidney disease, unique to the patients, because this organ is normal in LS-*G6pc^−/−^* mice.

A serious complication of GSDIa is renal damage and this is comparable to diabetic nephropathy. In fact, hyperlipidemia is considered to be a risk factor for the progression of diabetic nephropathy and correlates directly with the evolution of renal damage in patients with GSDI. In this respect, the control of hyperlipidemia is important to both prevent severe kidney damage and improve the efficacy of the current treatment. The identification of a set of microRNAs involved in kidney disease may thus be helpful for identifying new drugs to treat renal pathology in GSDIa patients.

In this study we have identified the Exo-miR signatures’ target genes and organized them into molecular pathways to gain information on the events potentially controlled by the exosome cargo. Thus, our data provide evidence that the Exo-miR profiles identified may relate to the specific affected organ gene expression and that the long-term consequences of GSDIa can be monitored through Exo-miRs assessment.

In conclusion, our results may evolve into protocols to counteract both the progression of liver degeneration leading to HCA and HCC onset as well as kidney disease and failure using circulating microRNAs as biomarkers.

## Figures and Tables

**Table 1:** Patient and healthy donors cohort characteristics.

Controls	Age (Years)	Gender	Tumor
CTR 01	10	M	no
CTR 02	2	M	no
CTR 03	6	M	no
CTR 04	4	F	no
CTR 05	12	F	no
CTR 06	16	F	no
CTR 07	14	F	no
CTR 08	18	M	no
CTR 09	21	M	no
CTR 10	38	F	no
CTR 11	16	F	no
CTR 12	61	F	no
CTR 13	52	F	no
CTR 14	48	M	no
Patients			
GSD 01	6	M	no
GSD 02	7	F	no
GSD 03	7	F	no
GSD 04	8	M	no
GSD 05	10	F	no
GSD 06	11	M	no
GSD 07	12	F	no
GSD 08	14	F	no
GSD 09	16	F	no
GSD 10	16	M	no
GSD 11	19	M	no
GSD 12	19	F	no
GSD 13	19	M	no
GSD 14	20	F	no
GSD 15	20	F	yes
GSD 16	21	F	no
GSD 17	22	F	no
GSD 18	22	M	no
GSD 19	22	M	yes
GSD 20	22	M	yes
GSD 21	22	F	no
GSD 22	24	F	no
GSD 23	26	F	yes
GSD 24	26	M	no
GSD 25	26	F	no
GSD 26	28	M	no
GSD 27	28	F	yes
GSD 28	29	M	no
GSD 29	30	M	yes
GSD 30	31	M	yes
GSD 31	31	F	yes
GSD 32	31	M	yes
GSD 33	32	M	yes
GSD 34	34	F	yes
GSD 35	35	F	no
GSD 36	36	F	yes
GSD 37	36	M	no
GSD 38	37	M	no
GSD 39	37	F	no
GSD 40	39	M	yes
GSD 41	40	F	yes
GSD 42	45	M	yes
GSD 43	49	F	yes
GSD 44	53	M	yes
GSD 45	63	F	yes

The table reports the main characteristics of the patients and the healthy donors enrolled in the study.

**Table 2:** Differentially expressed Exo-miRs in GSDIa patients.

Exo-miR ^a^	GSD HCA vs. GSD NO HCA ^b^	*p*-Value ^c^	GSD vs. CTRL ^c^	*p*-Value ^d^
miR-221-3p	2.46	0.01		
miR-195-5p	2.15	0.01		
miR-19a-3p	−0.64	0.006	1.15	0.01
miR-203-3p	−1.39	0.03		
miR-483-5p			2.17	0.0003
miR-454-3p			1.47	0.007
miR-122-5p			1.33	0.01
miR-342-3p			1.46	0.01
miR-376c-3p			1.20	0.01
miR-145-5p			−1.54	0.01
miR-103-3p			−1.11	0.03
miR-27b-3p			−1.01	0.04
miR-324-5p			−1.08	0.04
miR-150-5p	1.34	0.04		

^a^ MicroRNA identifiers were sorted by alphabetical order. Data were analyzed using the PIPE-T tool (Zanardi et al. 2019). MicroRNAs with a ^p^-value <0.05 and log2 fold change >0.58 or log2 fold change <−0.58 are considered significant. ^b^ Log2 fold change comparing the expression of microRNA between GSDIa patients with HCA and patients without HCA. Positive values indicate upregulation in patients with HCA. ^c^ Log2 fold change comparing the expression of microRNAs between GSDIa patients and healthy donors. WT mice. Positive values indicate upregulation in patients. ^d^ Significance of the differential expression according to RankProd method.

**Table 3:** Identification of microRNAs regulating genes expressing proteins differentially represented in the proteomic profile of the LS-*G6pc*^−/−^ mice.

Inflammatory and Immune Response		miR 27-b-3p	miR-103a-3p	miR-324-5p	miR-19a-3p	miR-145-5p	miR-203a-3p	miR-195-3p	miR-454-3p	miR-122-5p	miR-150-5p
CD163	acute-phase response (GO:0006953)				+						
MBL2	innate immune response (GO:0045087)				+	+	+				
C3	complement and coagulation cascades (map04610)	+									
C5	complement and coagulation cascades (map04610)				+						
**Glucose and Lipid Metabolism**											
ACACA	fatty acid biosynthetic process (GO:0006633)	+	+		+	+					
ACACB	fatty acid biosynthetic process (GO:0006633)		+	+	+	+					
FDPS	cholesterol biosynthetic process (GO:0006695)		+								
GOT2	2-oxocarboxylic acid metabolism (map01210)		+								
GPT	2-oxocarboxylic acid metabolism (map01210)		+			+					
HMGCS1	cholesterol biosynthetic process (GO:0006695)	+			+		+				
HSD17B7	cholesterol biosynthetic process (GO:0006695)						+				
IDH4	2-oxocarboxylic acid metabolism (map01210)		+								
LDHA	2-oxocarboxylic acid metabolism (map01210)		+								
MVD	cholesterol biosynthetic process (GO:0006695)		+								
TM7SF2	cholesterol biosynthetic process (GO:0006695)		+			+					
**Response to Hypoxia**											
LDHA,	pyruvate metabolism (map00620)		+				+				
PKLR	glycolytic process (GO:0006096)						+			+	
GAPDH	glycolytic process (GO:0006096)						+				+
DCN	homeostatic process (GO:0042592)		+								
FBP1	glycolytic process (GO:0006096)									+	
GBE1	glycogen biosynthetic process (GO:0005978)					+	+	+			
GLRX	homeostatic process (GO:0042592)	+									
NEDD4L	homeostatic process (GO:0042592)				+		+		+		
PGK1	glycolytic process (GO:0006096)	+	+		+		+	+	+	+	+
PLIN2	homeostatic process (GO:0042592)		+				+		+		
UGP2	glycogen biosynthetic process (GO:0005978)						+	+			
ALDOA	glycolytic process (GO:0006096)									+	
ALDOB	glycolytic process (GO:0006096)				+			+			
GALK1	glycolytic process (GO:0006096)										+
MIF	homeostatic process (GO:0042592)								+	+	
PCK1	homeostatic process (GO:0042592)							+	+	+	
S100A4	epithelial to mesenchymal transition (GO:0001837)										+

Protein sets related to inflammatory and immune response, glucose and lipid metabolism and response to hypoxia are shown and related pathway/process names and identifiers are indicated.

## Data Availability

The raw file from each array card experiment has been deposited at the Gene Expression Omnibus (GEO) public repository at NCBI (https://www.ncbi.nlm.nih.gov/geo (accessed on 31 May 2021)) and can be accessed through the GEO series accession number GSE188956.

## Supplementary Materials

The following are available online at https://www.mdpi.com/article/10.3390/ijms23010328/s1.
