# Supplementary material for: Exosomal MicroRNAs as Potential Biomarkers of Hepatic Injury and Kidney Disease in Glycogen Storage Disease Type Ia Patients
Source: Int J Mol Sci. 2021 Dec 28;23(1):328. doi: 10.3390/ijms23010328 (PMC8745197; doi:10.3390/ijms23010328)
Supplement: Supplementary file 1 [file ijms-23-00328-s001.zip › ijms-1493201-supplementary/SUPPLEMENTARY FILES Fig Legends.pdf]

SUPPLEMENTARY FILES

Figure S1. **Characterization of exosomes isolated from plasma of GSD1a patients**. Dynamic light scattering analysis shows the size distribution of isolated microvesicles from a representative GSD1a patient. The diameter (nanometers) is reported on X-axis, while the number of microvesicles is on the Y-axis.

Figure S2 - Qualitative and quantitative assessment of the noise reduction from GSDIa patients and CTRL subjects Exo-miRs expression profiles.

The box plots reported in panels A and B show the distribution of Ct and delta Ct values for every sample before and after data normalization, respectively. The plot reported in panel C shows the ECDFs (y axis) and the coefficient of variation (CV) for every sample before (blue line) and after (Green line) data normalization. Global mean was used to normalize the data. Kolmogorov-Smirnov test assessed the significance of the separation between the curves and the p-value is reported on top of the plot. P-value lower than 0.05 is considered significant.

Figure S3. Violin plots of the significantly modulated miR expression. X- axis reports the group label used to perform the comparison. The Y-axis reports the delta Ct value obtained after data imputation by PIPE-T tool. Dotted line indicates the zero value. The miR name and RankProd p-value is reported on top of the plot.
