# Supplementary figures and images for: Exosomal MicroRNAs as Potential Biomarkers of Hepatic Injury and Kidney Disease in Glycogen Storage Disease Type Ia Patients
Source: Int J Mol Sci. 2021 Dec 28;23(1):328. doi: 10.3390/ijms23010328 (PMC8745197; doi:10.3390/ijms23010328)

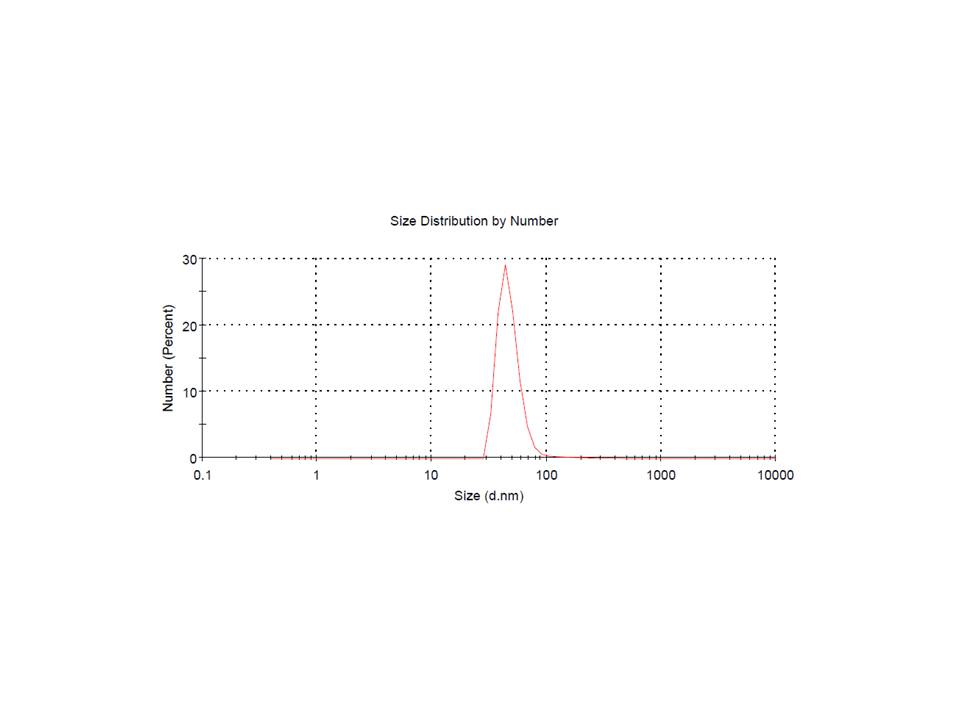

Supplement: Supplementary file 1 [file ijms-23-00328-s001.zip › ijms-1493201-supplementary/Figure S1_new.jpg]

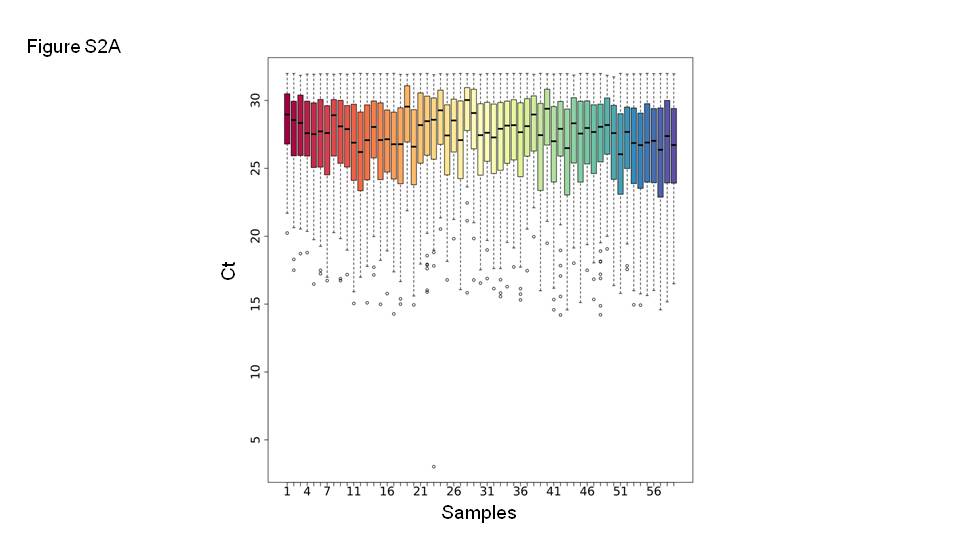

Supplement: Supplementary file 1 [file ijms-23-00328-s001.zip › ijms-1493201-supplementary/Figure S2A_high.jpg]

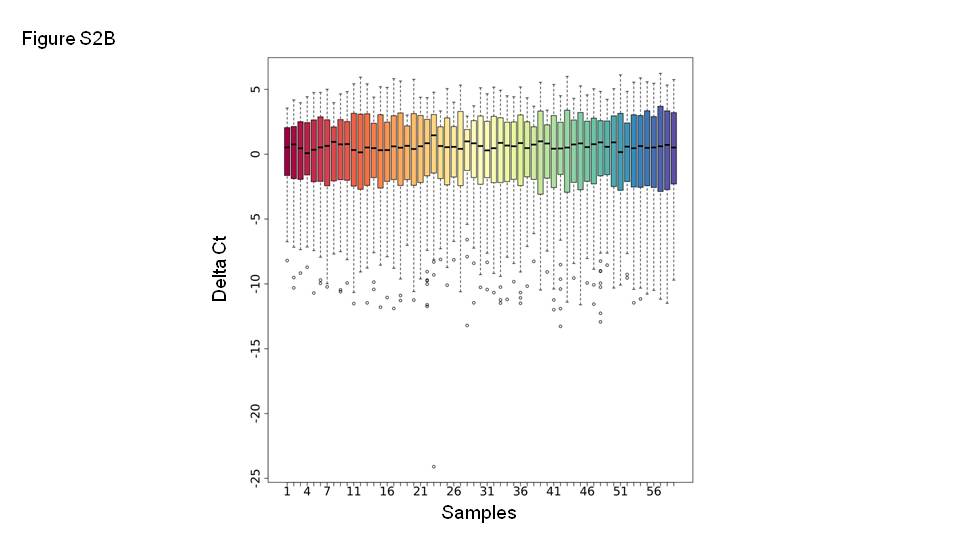

Supplement: Supplementary file 1 [file ijms-23-00328-s001.zip › ijms-1493201-supplementary/Figure S2B_high.jpg]

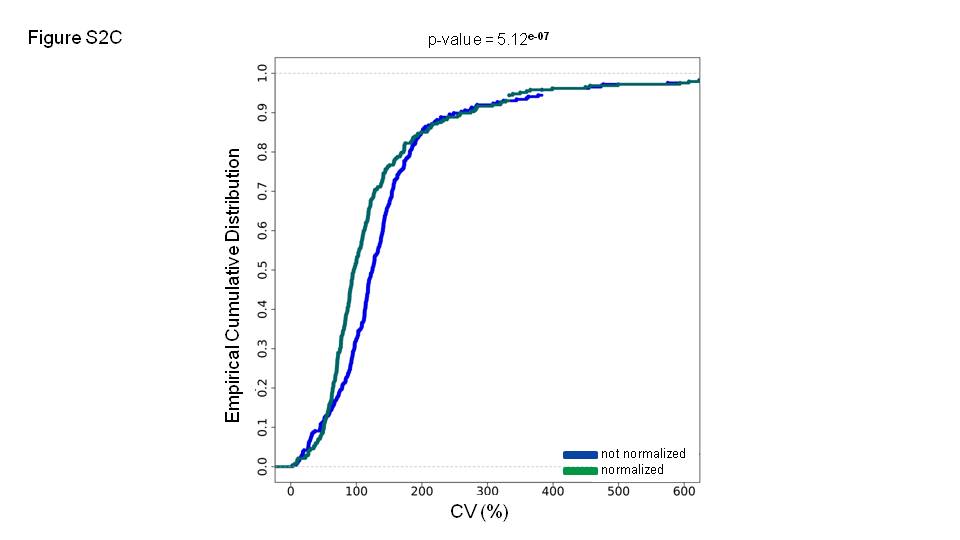

Supplement: Supplementary file 1 [file ijms-23-00328-s001.zip › ijms-1493201-supplementary/Figure S2C_high.jpg]

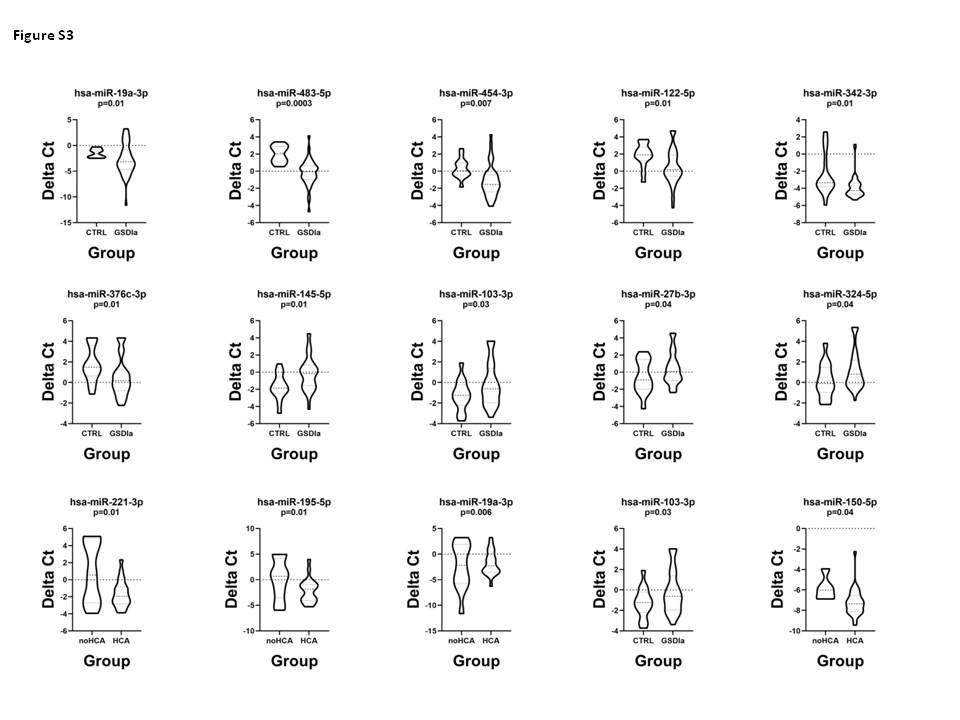

Supplement: Supplementary file 1 [file ijms-23-00328-s001.zip › ijms-1493201-supplementary/Figure S3_new.jpg]
